# Treatment of Heart Failure With Mid-Range Ejection Fraction: A Summary of Current Evidence

**DOI:** 10.3389/fcvm.2021.653336

**Published:** 2021-05-12

**Authors:** Teng Ma, Yang Su, Jing Song, Dachun Xu

**Affiliations:** ^1^Department of Cardiology, Shanghai Tenth People's Hospital, Tongji University School of Medicine, Shanghai, China; ^2^Department of Cardiology, The Second Affiliated Hospital and Yuying Children's Hospital, Wenzhou Medical University, Wenzhou, China

**Keywords:** heart failure, HFmrEF, HFPEF, treatment, review

## Abstract

Heart failure (HF) is a complex syndrome causing heavy burden in public health, and the modern objective assessment of it is based on the left ventricular ejection fraction (LVEF). In 2016, the European Society of Cardiology classified the “gray area” in HF with LVEF of 40–49% as a new HF phenotype (HFmrEF) in an attempt to uncover the specific characteristics and treatment of these patients, which might recover or worsen to HFpEF or HFrEF, respectively, or conversely from these two subtypes. Up to now, many studies have demonstrated that patients with HFmrEF would possibly gain more benefits from some targeted therapies with HFrEF than those with HFpEF. This review summarizes what is known about the findings in the treatment of HFmrEF and discusses what should be done to better define the peculiar HF phenotype in the future.

## Introduction

Heart failure (HF) is a complex clinical syndrome characterized by reduced cardiac output and/or elevated filling pressures at rest or with exertion. Recognizing different heart failure (HF) subtypes is so important, not only because it broadly frames differences in the underlying pathophysiology, but also because each of the HF subtypes presents different outcomes in therapeutic approaches (1, 2). The modern management of heart failure (HF) is primarily guided by clinical objective assessments of left ventricular ejection fraction (LVEF), which has been proven to be an efficacious predictive method of adverse outcomes even in patients without symptomatic HF.

In 2016, the European Society of Cardiology classified HF with mid-range ejection fraction (HFmrEF) as LVEF of 40–49% (3), which has often been considered a “gray” area in HF, as HFmrEF remains insufficiently characterized compared with the HFrEF and HFpEF subtypes in the past years. This new classification, as acknowledged in the guidelines, is an attempt to stimulate research and resolve critical clinical questions, rather than a true admittance of an independent phenotype different from the other groups. And as expected, there has been research on the clinical entity of HFmrEF in recent years, which presented us with expanding insights into epidemiology, pathophysiology, clinical characteristics, morbidity and mortality, and treatment for patients with HFmrEF. Clinical trial data suggest a HFmrEF prevalence of 14–24% among the overall HF population (4–8).

The EF may change with treatment and over time, and the heterogeneity is deduced by the different etiology of HF. A considerable number of patients transition to either HFrEF or HFpEF while on treatment. Coronary artery disease seems to be common, and it seems to play a critical role for worsening from HFpEF to HFmrEF or from HFmrEF to HFrEF. As there are a flurry of findings that HFmrEF specifies the aspects resembling the other two HF categories, which provide us with a feasible explanation of the controversies about why some researchers thought HFmrEF was a “transition phase” of HFrEF and HFpEF. This raises a question of which potential therapies thus far reserved for patients with HFrEF may be beneficial in those with intermediate LVEF.

As there are difficulties in the enrollment of patients with HFmrEF, there have been no randomized controlled trials (RCTs) dedicated to evaluate the effect of therapy. Therefore, we could only find some information on the overlap between HFmrEF and other groups, as we did from the CHARM, TOPCAT, and PARAGON clinical trials, which all showed an effect of different drugs in the lower end of the LVEF spectrum included in these studies, such as 40–50% or 45–50%. We have made some progress in understanding the treatment efficacy of neurohormonal antagonists, including angiotensin-converting enzyme inhibitors/ACEI, angiotensin receptor blockers/ARB, beta-blockers, and mineralocorticoid receptor antagonists/MRA, in patients with HFmrEF. In this review, we will present an overview about the updated therapies for patients with HFmrEF.

## Angiotensin-Converting Enzyme Inhibitors (ACEI) and Angiotensin Receptor Blockers (ARB)

In a *post-hoc* analysis of a randomized clinical trial, named the CHARM program, in which the 7,598 patients with available integer digit EF were divided into three parts: HFrEF, HFmrEF, and HFpEF (9), the authors evaluated the treatment effect of candesartan in patients with HFmrEF, and found there was a smaller risk of primary outcome [HR: 0.76; 95% CI (0.61–0.96); *p* = 0.02] and recurrent HF hospitalization [HR: 0.48, 95% CI (0.33–0.70), *p* < 0.001] in the treatment group during the mean follow-up of 2.9 years. It is notable that the treatment efficacy of candesartan was constant at a lower EF and generally began to decline at EF > 50%. However, in the randomized controlled I-PRESERVE trial with LVEF >45% (10), there was no difference between the irbesartan treatment group compared with the placebo group, though the average LVEF was higher in this trial (mean LVEF, 59%) compared with the CHARM-preserved trial (mean LVEF, 54%). In an observational study (11), the OPTIMIZE-HF trial, HF patients with LVEF >40% also did not benefit from ACEI/ARB therapy in the first 60 to 90 days of follow-up.

## Beta-Blockers

Cleland et al. (12) used a meta-analysis of randomized controlled trials to demonstrate that beta-blockers may reduce CV death in HFmrEF patients in sinus rhythm compared with placebo [HR 0.48; 95% CI (0.24–0.97); *p* = 0.04] and improve left ventricular systolic function with a higher LVEF using data from double-blind, randomized, placebo-controlled trials. Similar to the outcomes above, several observational studies suggested that beta-blockers treatment may have benefits in cardiovascular outcomes in the HFmrEF population. In the multicenter prospective registry CHART-2 cohort (13), beta-blocker use was associated with reduced mortality among those with HFmrEF. Similarly, in the Swedish Heart Failure Registry (6), beta-blockers were associated with reduced mortality only in the presence of CVD (HR up to 1 year 0.74, 95% CI 0.59–0.92), nevertheless, ACEI/ARBs and statins were associated with lower 1-year all-cause mortality with or without CVD. However, in the OPTIMIZE-HF trial (14), initiation of beta-blockers did not show improved outcomes in the HF patients with LVEF >40%, and another study also revealed that there were no improvements in all-cause mortality in those with EF >40% (15).

## Mineralocorticoid Receptor Antagonists (MRAs)

A study used data from a randomized controlled trial called TOPCAT (16) to assess the relationship between efficacy and outcome of spironolactone and LVEF, found that LVEF modified the treatment efficacy of spironolactone, those with LVEF between 45 and 50% had a lower primary outcome [HR 0.72, 95% CI (0.50, 1.05)] and HF hospitalization [HR 0.76, 95% CI (0.46, 1.27)]. Along with this, in a prospective study (17), during a mean follow-up of 2.2 years, Enzan et al. found that patients with spironolactone had a lower incidence rate of primary outcome (all-cause death and or HF rehospitalization) than those without it [RR 0.61, 95 CI (0.44–0.86), *P* = 0.004].

## Sacubitril/Valsartan

There were 4,822 patients with LVEF >45% who were randomly assigned to sacubitril/valsartan or valsartan groups in the PARAGON-HF trial (18). The primary composite outcome of total hospitalizations for heart failure and death from cardiovascular causes had no statistical significance between the two groups. Although statistically non-significant, it is noticeable that sacubitril/valsartan had a lower rate of hospitalization for heart failure than valsartan alone (rate ratio 0.87, 95%CI 0.75–1.01, *p* = 0.06). And of the 12 pre-specified subgroups, two showed a benefit in patients with an ejection fraction in the lower part (45–57%) of the study and in women. Along with this, Solomon and colleagues (19) combined data from the PARADIGM-HF (LVEF eligibility ≤ 40%; *n* = 8,399) and PARAGON-HF trials, as the two studies had similarities in many aspects such as eligibility criteria, similar control groups (enalapril or valsartan, respectively), and outcome assessment. The pooled analysis containing a total cohort of 13,195 patients suggested that patients with LVEF lower than normal, including HFmrEF or borderline ejection fraction, would possibly benefit, particularly in the combined end-point of cardiovascular mortality and first hospitalization for HF, from sacubitril/valsartan compared with RAS inhibition. And these therapeutic benefits appeared to extend to a higher LVEF range in women compared with men. A study suggested that combination use of sacubitril/valsartan rather than valsartan alone with MRA appeared to be associated with a lesser decline of renal function and no increase in severe hyperkalemia in patients with LVEF >45% in the PARAGON-HF trial, which would provide us with a new insight of benefit of combined therapy (21). In the PARALLAX trial (22), randomizing 2,572 patients with a LVEF of >40%, NT-proBNP was significantly reduced in the sacubitril/valsartan group after 12 weeks vs. individualized medical therapy, and was associated with a reduction of left atrial size. But it is noticeable that there was a significantly difference in terms of NTproBNP cut-off diastolic dysfunction analysis and comorbidities compared with other trials. There was a lower risk of worsening renal function with sacubitril/valsartan in HF patients with LVEF >40% than those with LVEF ≤ 40% (23), according to a systematic review and meta-analysis of randomized controlled trials. Excitingly, the FDA panel has supported the expanded indication for sacubitril/valsartan, which would allow it to be a treatment for certain patients with HFpEF, and it is possible that sacubitril/valsartan would be efficacious in those with HFmrEF.

## Other Therapeutics

Digoxin had an intermediate effect on HFmrEF [HR: 0.80, 95% CI (0.63–1.03)] compared with HFrEF and HFpEF, and did not significantly reduce HF hospitalization in the HFmrEF population (20). Diuretics seem to be negatively associated with improved mortality in HFmrEF (13). Sodium glucose cotransporter 2 inhibitors (SGLT-2I), an inhibitor of a new pathway of HF treatment different from the neurohormonal one, are associated with reduced HF hospitalizations and CV death in patients with type 2 diabetes mellitus regardless of history of HF (24, 25), and the ongoing EMPEROR-Preserved, DELIVER, and SOLOIST-WHF trials may confirm the effect of these drugs on HF outcomes in patients with LVEF >45%. The summary of the effect of the main HF therapies on outcomes specifically in the HFmrEF population is reported in Table 1.

**Table 1:** Overview of the main studies investigating patients with HFmrEF.

**References**	**Study type**	**Inclusion criteria**	**LVEF**	**Patient number**	**Outcome for HFmrEF**
Lund et al. (9)	*Post-hoc* analysis of randomized trial	Patients enrolled in CHARM program	Full spectrum	7,599	Primary outcome for candesartan vs. placebo: [HR: 0.76, 95% CI (0.61, 0.96), *p* = 0.02]; recurrent HF hospitalization: [HR: 0.48, 95% CI (0.33, 0.70), *p* < 0.001]
Solomon et al. (16)	*Post-hoc* analysis of randomized trial	Patients with HF and LVEF ≥45% enrolled in TOPCAT	>45%	3,444	Primary outcome for spironolactone vs. placebo: [LVEF <50%,HR: 0.72, 95% CI (0.50, 1.05), *p* = 0.046]; heart failure hospitalization [LVEF <50%, HR: 0.76, 95% CI (0.46, 1.27), *p* = 0.039]
Cleland et al. (12)	Meta-analysis of randomized controlled trials	Included all patients with baseline LVEF and an electrocardiogram (ECG) that showed either sinus rhythm or AF/atrial flutter	Full spectrum	14,262	Beta-blockers may reduce CV death in HFmrEF patients in sinus rhythm compared with placebo [HR: 0.48, 95% CI (0.24, 0.97), *p* = 0.04]
Solomon et al. (18)	*Post-hoc* analysis of randomized trial	Patients with HF and LVEF ≥45% enrolled in PARAGON-HF	>45%	4,822	Primary events for sacubitril–valsartan vs. valsartan: [RR: 0.87, 95% CI (0.75, 1.01), *p* = 0.06]
Abdul-Rahim et al. (20)	*Post-hoc* analysis of randomized trial	Patients enrolled in DIG. HF patients with LVEF ≤ 45% and were in normal sinus rhythm (6,800 patients). HF patients with LVEF >45% were enrolled in an ancillary trial (988 patients)	Full spectrum	7,788	Digoxin had an intermediate effect in HFmrEF [HR: 0.80, 95% CI (0.63, 1.03)] compared with HFrEF and HFpEF; the composite of HF death or HF hospitalization [HR: 0.83, 95% CI (0.66, 1.05)]
Massie et al. (10)	Randomized controlled trial	Patients with HF and LVEF ≥45% in I-PRESERVE	≥45%	4,128	The primary outcome in the irbesartan group vs. the placebo group: [HR: 0.95, 95% CI (0.86, 1.05), *p* = 0.35); the secondary outcome: rates of death from any cause in the irbesartan group and the placebo group: [HR: 1.00, 95% CI (0.88, 1.14), *p* = 0.98]; rates for protocol-specified hospitalization: [HR: 0.95, 95% CI (0.85, 1.08), *p* = 0.44]
Enzan et al. (17)	Multicenter prospective registry	Patients with HF and with LVEF of ≥40 and <50% from JCARE-CARD	40–50%	457	Primary outcome for spironolactone vs. placebo: [IRR: 0.61, 95% CI (0.44, 0.86); *p* = 0.004]; composite of all-cause death or HF rehospitalization [adjusted HR: 0.63, 95% CI (0.44, 0.90), *P* = 0.010]
Tsuji et al. (13)	Multicenter prospective registry	Patients with HF and LVEF ≥45% enrolled in CHART-2	Full spectrum	3,480	Beta-blockers were positively associated with HFmrEF [HR: 0.57, 95% CI (0.37, 0.87), *p* = 0.010]; diuretics were negatively associated with improved mortality in HFmrEF [HR: 2.01, 95% CI (1.24, 3.28), *p* = 0.004]
Fonarow et al. (11)	Prospective registry	Patients with HF and LVEF ≥40% and left ventricular systolic dysfunction (LVSD) with reduced EF enrolled in OPTIMIZE-HF	≥40%	41,267	60- to 90-day mortality: [HR: 1.141, 95% CI (0.812, 1.603), *p* = 0.447] and rehospitalization rates [HR: 0.909, 95% CI (0.692, 1.196), *p* = 0.497] for ACEI/ARB; 60- to 90-day mortality: [HR: 1.209, 95% CI (0.872, 1.875), *p* = 0.255] and rehospitalization rates [HR: 0.923, 95% CI (0.723, 1.179), *p* = 0.523] for beta-blockers
Lund et al. (9)	Nationwide prospective registry	Patients with HF enrolled in SwedeHF	Full spectrum	51,060	Beta-blockers use and 1-year mortality in HFmrEF: mortality was reduced in HFmrEF with CAD [HR up to 1 year 0.74, 95% CI (0.59, 0.92)] but not in HFmrEF without CAD [HR 0.99, 95% CI (0.78, 1.26)]; angiotensin-converting enzyme inhibitors (ACEI)/angiotensin receptor blockers (ARBs)/statins were associated with reduced risk in all HFmrEF groups with or without CAD (all *p* ≤ 0.004)

*HF, heart failure; LVEF, left ventricular ejection fraction; HFmrEF, heart failure with mid-range ejection fraction; HFpEF, heart failure with preserved ejection fraction; HFrEF, heart failure with reduced ejection fraction; HR, hazard ratio; CI, confidence interval; CAD, coronary artery disease; IRR, incidence rate ratio; AF, atrial fibrillation; ACEI, angiotensin-converting enzyme inhibitors; ARBs, angiotensin receptor blockers*.

HFmrEF is not a stable phenotype, but a heterogenous condition with variable evolutions, which is proven by the fact that without any change in underlying pathophysiology, a number of HF patients move in and out of the HFmrEF range on serial echocardiograms (6, 26). The treatment and management of coronary artery disease and atrial fibrillation seems to be important in the process of heart failure phenotype transition. As indicated by the HF Long-Term Registry of the European Society of Cardiology (27), the prevalence of AF was higher with increasing LVEF (27% in HFrEF, 29% in HFmrEF, and 39% in HFpEF) and AF was associated with worse outcomes (combined HF hospitalization and all-cause mortality) in HFpEF [HR = 1.36, 95% CI (1.15–1.62), *p* < 0.001] and HFmrEF [HR = 1.30, 95% CI (1.06–1.61), *p* = 0.014], but not in HFrEF [HR = 0.96, 95% CI (0.84–1.09), *p* = 0.502]. In the SwedeHF trial (6), HFmrEF resembled HFrEF most notably for CAD (HFrEF 54%, HFmrEF 53%, HFpEF 42%, *p* < 0.001), and notable adjusted odds ratios (ORs) were similar for CAD [HFmrEF vs. HFpEF: OR 1.52, 95% CI (1.41–1.63); HFmrEF vs. HFrEF: OR 0.94, 95% CI (0.88–1.00)]. Although targeting patients of HFmrEF specifically, like we did in other HF groups, is efficacious for resolving questions that disturbed us, many have failed due to the difficulties in patient enrollment. In addition, the variability of LVEF measurements based on echocardiography influences the accuracy of EF evaluation. Potential solutions to these issues might include the following: (1) expanding the EF range of HFrEF and/or HFpEF to include HFmrEF or the entire EF spectrum, as we did in some research evaluating ARB, MRA, and ARNI therapy in HFmrEF, and (2) evaluating EF in a dynamic and serial way, as beyond evaluating baseline LVEF, the implications of longitudinal LVEF are becoming more important.

In the era of precision medicine, the future management of HFmrEF or HF patients may involve accurately evaluating cardiac function and identifying features of each patient with HF, which might provide us with more information about how to scientifically stratify risk factors and choose appropriate therapies beyond what is predicted by LVEF alone, help doctors discern true myocardial recovery from myocardial remission which includes reverse cardiac remolding, but the absence of signs of complete reversal of damage, and multiparametric approaches, such as biomarkers and image parameters, should be taken into account for the discovery of new more effective treatments.

## Conclusion

The expanding insights of HFmrEF indicate to us that it is an intermediate phenotype between HFrEF and HFpEF in terms of baseline characteristics, outcomes, and prognosis, but mildly resembles more that of the HFrEF subtype than HFpEF. As summarized in Table 2, ARB, beta-blockers, MRA, and ARNI may have potential cardiovascular benefits for patients with HFmrEF, but it is uncertain whether ACEI or SGLT-2I has cardiovascular benefits. Future research, especially RCTs, is needed to explore the expanding insights into this peculiar phenotype and to identify strategies that will best achieve improvements in cardiovascular outcomes.

**Table 2:** Medical therapy in heart failure.

	**ACEI**	**ARB**	**Beta-blocker**	**MRA**	**ARNI**	**SGLT2I**	**Diuretic**
HFrEF	↑↑	↑↑	↑↑	↑↑	↑↑	↑↑	**?**
HFmrEF	**?**	↑	↑	↑	↑	**?**	**?**
HFpEF	**×**	↑	**×**	↑	↑	**?**	**?**

*↑↑: Proven cardiovascular benefit*.

*↑: Potential cardiovascular benefit*.

***×**: No cardiovascular benefit*.

***?**: Uncertain cardiovascular benefit*.

*ACEI, angiotensin-converting enzyme inhibitors; ARB, angiotensin receptor blocker; MRA, mineralocorticoid receptor antagonist; ARNI, angiotensin receptor—neprilysin inhibitor; SGLT2I, sodium glucose cotransporter 2 inhibitors*.

## Author Contributions

All authors listed have made a substantial, direct and intellectual contribution to the work, and approved it for publication.

## Conflict of Interest

The authors declare that the research was conducted in the absence of any commercial or financial relationships that could be construed as a potential conflict of interest.
